# Procalcitonin as a diagnostic marker of ventilator-associated pneumonia in cardiac surgery patients

**DOI:** 10.3892/etm.2015.2175

**Published:** 2015-01-09

**Authors:** JIA JIAO, MIN WANG, JIANFENG ZHANG, KANGJUN SHEN, XIAOBO LIAO, XINMIN ZHOU

**Affiliations:** 1 Department of Cardiothoracic Surgery, The Second Xiangya Hospital, Central South University, Changsha, Hunan 410011, P.R. China; 2 Department of Cardiothoracic Surgery, The First Affiliated Hospital of Chongqing Medical University, Chongqing 400016, P.R. China

**Keywords:** procalcitonin, ventilator-associated pneumonia, cardiac surgery, diagnostic marker

## Abstract

The aim of the present study was to assess whether procalcitonin (PCT) can be used as a diagnostic marker for ventilator-associated pneumonia (VAP) in cardiac surgery patients. Between January 2012 and June 2013, a total of 92 patients were recruited and divided into non-VAP (59 patients) and VAP (33 patients) groups. The preoperative and postoperative characteristics of the patients were recorded. Serum levels of PCT, interleukin (IL)-6 and C-reactive protein (CRP) were measured using an electrochemiluminescence immunoassay. Subsequently, receiver operating characteristic curves of the PCT, IL-6 and CRP levels were constructed. In addition, associations between the sequential organ failure assessment (SOFA) scores and the serum levels of PCT, IL-6 and CRP in the VAP patients were analyzed. No statistically significant difference was observed between the non-VAP and VAP patients in the occurrence of postoperative complications. However, the SOFA scores (days 1 and 7), the duration of stay in the intensive care unit and the mechanical ventilation time were all significantly higher in the VAP group when compared with the non-VAP group (P<0.05). The optimum PCT cut-off value for VAP diagnosis on day 1 was 5.0 ng/ml, with a sensitivity of 91% and a specificity of 71%. The serum PCT levels on days 1 and 7 were found to correlate positively with the SOFA scores (r=0.54 and r=0.66 for days 1 and 7, respectively). Therefore, the results suggested that serum PCT may be used as diagnostic marker for VAP in patients following cardiac surgery.

## Introduction

Major cardiac surgery patients are a particularly high-risk population for nosocomial infections (1). Ventilator-associated pneumonia (VAP) is the primary infectious complication for cardiac surgery patients (2) and is associated with a marked increase in morbidity and mortality rates (3). The prevalence of VAP following cardiac surgery is estimated to be 17.9% in patients who receive mechanical ventilation for >48 h and 28.8% in patients who undergo mechanical ventilation for >72 h (4). The median mechanical ventilation time and duration of hospital stay are significantly prolonged in patients who develop VAP following cardiac surgery (5). Tamayo *et al* determined that cardiac surgery patients who develop VAP have a mortality risk that is 8.53 times higher than those without VAP (6). In addition, the authors showed that VAP is the most important independent risk factor for mortality following major cardiac surgery. Therefore, early prevention, diagnosis and treatment of VAP is essential.

Procalcitonin (PCT), a protein of 116 amino acids with a molecular weight of 13 kDa, is the precursor molecule of calcitonin. PCT is usually produced by parafollicular cells in the thyroid gland (7). Since PCT levels were found to be elevated in patients with bacterial infection, increasing interest has been directed at the possibility of using PCT as an indicator of infection (7–9). In addition, PCT has been shown to be useful in discriminating between septic and nonseptic inflammation and may be a promising biomarker for the diagnosis of VAP (10). However, cardiac surgery and the use of cardiopulmonary bypass (CPB) may activate the immune system and lead to a systemic inflammatory response, which increases the difficulty of identifying infectious complications in cardiac surgery patients. Furthermore, CPB and noninfectious complications, such as organ dysfunction/failure, contribute to increased PCT levels (11). Therefore, the value of PCT as a diagnostic marker of infection in cardiac surgery remains controversial. Adib-Conquy *et al* (12) showed that PCT is not specifically a marker for infection, as PCT levels may increase markedly in cases of acute inflammation with no infection. In addition, Sinning *et al* (13) suggested that systemic inflammatory response syndrome (SIRS) is associated with PCT in patients with a transcatheter aortic valve implantation. Therefore, the feasibility of using PCT as a diagnostic marker for VAP in cardiac surgery patients remains unclear and requires further study.

The present study examined 92 patients who received ≥48 h of mechanical ventilation following cardiac surgery. The patient serum levels of PCT, interleukin (IL)-6 and C-reactive protein (CRP) were analyzed using an electrochemiluminescence immunoassay (ECLIA). The incidence and type of postoperative noninfectious complications in the patients were also recorded. In addition, the diagnostic values of serum levels of PCT, IL-6 and CRP were analyzed by linear regression.

## Materials and methods

### Patients

The study was conducted at the intensive care unit (ICU) of the Department of Cardiothoracic Surgery at the Second Xiangya Hospital of Central South University (Changsha, China). In total, 92 consecutive patients (age, ≥18 years) who had received ≥48 h of mechanical ventilation were recruited between January 2012 and June 2013. All the patients had undergone elective cardiac surgery with CPB. Patients were excluded from the study if they had been diagnosed with pneumonia prior to the mechanical ventilation, were pregnant, had received immunosuppressants or long-term corticosteroid therapy or had a coexisting extrapulmonary infection that required antibiotic therapy for more than three days prior to or following the cardiac surgery. The patients were divided into VAP and non-VAP patient groups.

The study protocol was approved by the Ethics Committee of the Second Xiangya Hospital of Central South University, and patients provided written informed consent.

### Diagnosis of VAP and classification of complications

The study focused on patients in the early stages of VAP that had been diagnosed between 48 h and six days after initiation of the mechanical ventilation. Diagnosis of VAP (14) was determined using a novel criteria, based on chest X-ray results, with at least two of the following features: Fever with a body temperature of >38°C; a white blood cell count of >11,000 or <3,000/μl; or the presence of purulent endotracheal secretions. Microbiological samples were obtained by fibroscopic bronchoalveolar lavage (BAL), and the growth of ≥10^4^ colony-forming units/ml microorganism culture of BAL was considered to be positive.

A number of noninfectious complications were defined according to specific criteria. Cardiovascular complications were defined as a low cardiac output with a cardiac index of <2.0 l/min/m^2^, postoperative myocardial infarction and malignant ventricular arrhythmia (sustained and requiring further treatment). Pulmonary dysfunction was diagnosed if the PaO_2_/FiO_2_ ratio was <200 mmHg, unless this was caused by cardiogenic factors. Acute renal failure was defined as requiring temporary hemofiltration or hemodialysis, while neurological complications included stroke and ischemic cerebral infarction. Finally, rethoracotomy due to bleeding was a potential noninfectious complication.

### Data collection

Follow-up lasted for seven days or until patient mortality. Patient characteristics and clinical data, including the age, gender, preoperative diagnosis, left ventricular ejection fraction, brain natriuretic peptide level, type of surgery, CPB time, aortic cross-clamp time, mechanical ventilation time, length of ICU stay and diagnosis of noninfectious complications, were collected.

### Biological measurements and sequential organ failure assessment (SOFA)

Blood samples were collected prior to cardiac surgery in order to define the baseline biological measurement values. Patients were evaluated daily for symptoms of VAP. Once VAP was diagnosed (defined as day 1), the patients received empirical antibiotic treatment until the results of the bacterial culture were available. In the non-VAP group, day 1 was defined as the day when the tracheal intubation was removed from each patient. The levels of PCT, IL-6 and CRP were measured using an ECLIA (Roche Diagnostics GmbH, Mannheim, Germany) for the patients in the VAP and non-VAP groups on days 1, 3, 5 and 7.

SOFA was conducted on days 1 and 7 for the patients in the VAP and non-VAP groups (15).

### Statistical analysis

Data are presented as the mean ± standard deviation and statistical analysis was performed using SPSS 16.0 software (SPSS, Inc., Chicago, IL, USA). Comparisons between the groups were conducted using the Student’s t-test, the Mann-Whitney test or Fisher’s exact method. Receiver operating characteristic (ROC) curves were used to determine the optimum threshold values for sensitivity and specificity. Correlations were analyzed using Pearson’s correlation coefficient. Two-tailed P-values of <0.05 were considered to indicate a statistically significant difference.

## Results

### Preoperative and postoperative patient characteristics

A total of 92 patients, including 59 non-VAP patients and 33 VAP patients, were included in the prospective cohort study. Detailed preoperative and postoperative characteristics of the VAP and non-VAP patients are summarized in Table I. There were no statistically significant differences in preoperative characteristics between the VAP and non-VAP group patients. In addition, no statistically significant difference was observed in the occurrence of postoperative complications between the VAP and non-VAP patients (33/33 vs. 53/59, P=0.058). There were six patients in the VAP group and four in the non-VAP group who suffered from multiple noninfectious postoperative complications. The SOFA scores on days 1 and 7 were significantly higher in the VAP group when compared with the non-VAP group (P<0.001). Furthermore, the durations of ICU stay (P=0.001) and mechanical ventilation (P<0.001) were longer in the VAP group when compared with the non-VAP group.

### Serum PCT, CRP and IL-6 levels

**Figure 1:**
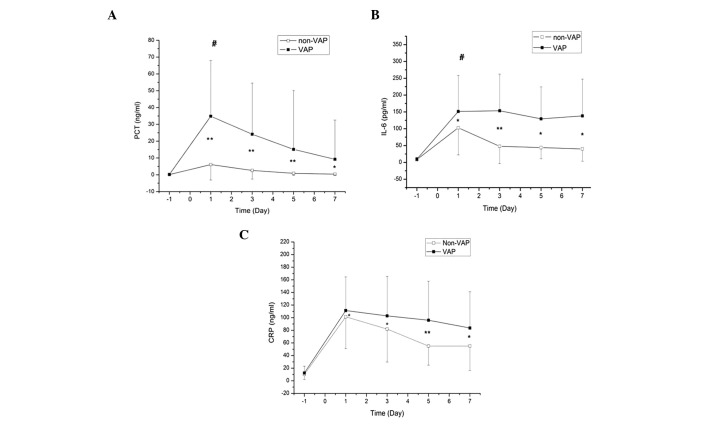
Comparison of the serum concentrations of (A) PCT, (B) IL-6 and (C) CRP between the VAP and non-VAP groups preoperatively and on days 1, 3, 5 and 7. ^*^P<0.05 and ^**^P<0.01, vs. preoperative levels in both VAP and non-VAP groups; ^#^P<0.05, vs. non-VAP group on day 1. PCT, procalcitonin; IL-6, interleukin-6; CRP, C-reactive protein; VAP, ventilator-associated pneumonia.

Preoperative and postoperative (days 1, 3, 5 and 7) serum PCT, IL-6 and CRP levels were compared between patients in the VAP and non-VAP groups (Fig. 1). No statistically significant differences were observed in the preoperative serum levels of PCT, IL-6 and CRP between the VAP and non-VAP groups (P>0.05). However, the serum levels of PCT, IL-6 and CRP in the VAP and non-VAP groups were significantly higher on days 1, 3, 5 and 7 when compared with the preoperative baseline levels (P<0.05). Levels of PCT and IL-6 on day 1 were significantly higher in the VAP group when compared with the non-VAP group (P<0.05), while there was no statistically significant difference in the CRP levels between the groups on day 1 (P>0.05).

### Diagnostic value of serum PCT, IL-6 and CRP levels

**Figure 2:**
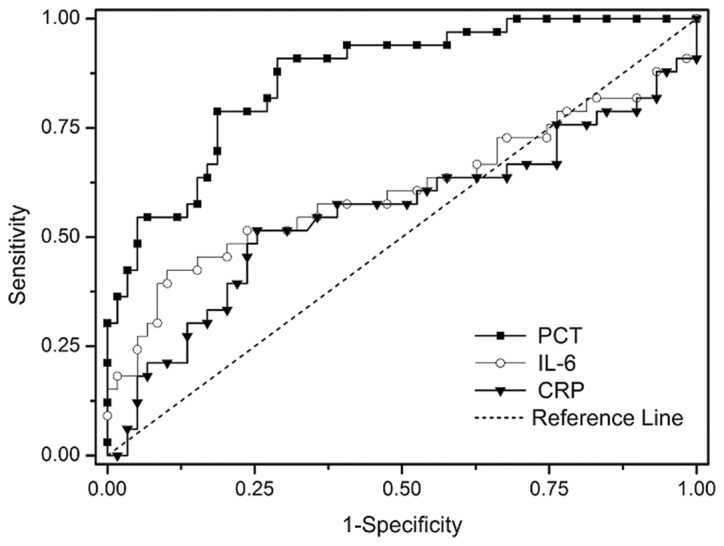
Receiver operating characteristic curves of serum PCT, IL-6 and CRP on day 1. PCT, procalcitonin; IL-6, interleukin-6; CRP, C-reactive protein.

Using the ROC curves, it was concluded that the optimum PCT cut-off value for VAP diagnosis on day 1 was 5.0 ng/ml, with a sensitivity of 91% and a specificity of 71%. The areas under the ROC curve were 0.87, 0.61 and 0.56 for PCT, IL-6 and CRP, respectively (Table II; Fig. 2).

### Linear correlation analysis

**Figure 3:**
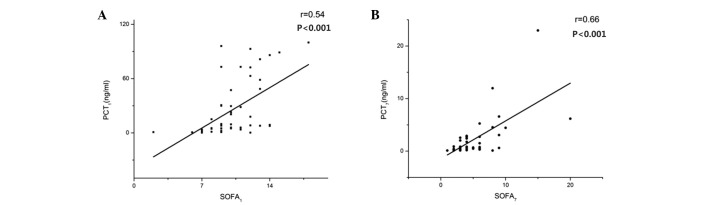
Linear correlation analysis between the levels of serum PCT in the VAP patients and the corresponding SOFA score on (A) day 1 and (B) day 7. PCT, procalcitonin; SOFA, sequential organ failure assessment.

Associations between the serum levels of PCT, IL-6 and CRP in the VAP patients and the corresponding SOFA scores on days 1 and 7 were analyzed using linear correlation analysis. The PCT levels on days 1 and 7 correlated positively with the SOFA scores (r=0.54, P<0.001 and r=0.66, P<0.001, respectively; Fig. 3), while the levels of IL-6 and CRP were not found to correlate with the SOFA scores (P>0.05). There were no statistically significant linear correlations between the serum levels of PCT, IL-6 and CRP (P>0.05).

## Discussion

Although numerous studies have reported the use of serum PCT as a marker for VAP (16–18), the results have been contradictory. In the present study, the serum levels of PCT, IL-6 and CRP were measured in VAP and non-VAP cardiac surgery patients. The optimum PCT cut-off value for VAP diagnosis on day 1 was 5.0 ng/ml, with a sensitivity of 91% and a specificity of 71%. Furthermore, the serum levels of PCT were shown to correlate positively with the SOFA scores on days 1 and 7. Therefore, serum PCT may be used as a diagnostic marker for VAP in patients following cardiac surgery.

Duflo *et al* (16) found that serum levels of PCT increased significantly in VAP patients when compared with non-VAP patients until day 3. The optimum cut-off value of serum PCT for the diagnosis of VAP was 3.9 ng/ml, with a low sensitivity of 41%, but an excellent specificity of 100%. Oppert *et al* (17) studied 28 patients who exhibited a spontaneous return in circulation following cardiac arrest and determined the optimum serum PCT cut-off value to be 1 ng/ml, with a sensitivity of 100% and a specificity of 75% for VAP diagnosis. However, a study of 73 suspected VAP patients by Luyt *et al* (18) found PCT to be a poor marker for VAP, with a sensitivity of 72% and a specificity of only 24%, at a cut-off level of 0.5 ng/ml. Mixed ICU, early- or late-onset of VAP and the use of antibiotics in advance of diagnosis may lead to decreased sensitivity or specificity. In the study by Luyt *et al*, preinfected patients were not excluded, while in the study by Oppert *et al*, only 10 cases among 12 patients exhibited early-onset VAP. Furthermore, the population assessed in the study by Duflo *et al* consisted of late-onset VAP patients who had received over two years of ventilation. These factors may have caused underestimation or overestimation of the diagnostic value of PCT, which may account for the discrepancies among previous findings. In the present study, all the patients exhibited early-onset VAP following cardiac surgery, which ensured that the subjects were selected from a homogeneous patient population.

Numerous studies have shown that cardiac surgery and CPB may affect the serum levels of various biomarkers, including PCT, CRP, IL-6 and IL-8 (19,20). Among these proteins, PCT in particular is considered to be closely associated with infection conditions. In the absence of a bacterial infection, SIRS caused by surgery or CPB is an important factor in the stimulation of PCT expression (13). Cardiac surgery and CPB result in the exposure of blood vessels to nonphysiological surfaces, the translocation of endotoxin and the release of cytokines, such as IL-6, all of which have been shown to induce PCT (13). However, numerous studies (20–22) have indicated that the CPB procedure itself causes only a moderate, transient postoperative increase in PCT levels. PCT levels peaked on days 1 or 2 following surgery and decreased continuously to a normal value, with the peak value not generally exceeding 2 ng/ml (22). Therefore, Jebali *et al* suggested that the diagnostic properties of PCT may not be evident during the first two days following surgery (23). Diagnosis of VAP using PCT as a biomarker requires the patient to undergo mechanical ventilation for >48 h. However, all the subjects in the present study were postoperative patients who had been using a tracheal intubation for over two days; thus, the CPB-induced postoperative peak in PCT levels was avoided. Therefore, this experiment did not consider the impact of mechanical ventilation time.

Markedly increased levels of PCT are primarily associated with postoperative complications, including low cardiac output syndrome, perioperative myocardial infarction, pulmonary dysfunction, renal failure and infection (23). Prat *et al* (24) found that PCT levels were significantly higher in 15 patients with infectious or noninfectious complications compared with patients who recovered without complications. The optimum cut-off value of PCT on the first postoperative day was 2 ng/ml, with a sensitivity of 92.3% and a specificity of 93.8%. Sponholz *et al* (11) suggested that PCT levels were significantly higher in patients with postoperative infection when compared with those in noninfected patients. Therefore, continuous monitoring of the serum PCT levels was the most effective approach for diagnosing postoperative infection.

Although the observation period began after >48 h of postoperative intubation in the present study, there remained 44/59 patients with PCT levels of >2 ng/ml in the non-VAP group, and all the patients in the VAP group had PCT levels of >2 ng/ml (33/33). According to the aforementioned criteria, all the patients in the VAP group exhibited noninfectious complications and there were no statistically significant differences when compared with the non-VAP group (53/59). However, the SOFA scores of the patients in the VAP group were notably higher when compared with the non-VAP group. A previous study demonstrated that higher SOFA scores were associated with significantly higher serum PCT concentrations during the course of multiple organ dysfunction syndrome (MODS) and sepsis (25). In addition, Haasper *et al* (26) showed that the presence of significantly higher levels of PCT in patients resulted in the development of MODS. Differences in SOFA scores may increase the levels of PCT in the VAP group to a greater extent compared with the non-VAP group, which may indirectly increase the diagnostic accuracy of VAP. In the present study, the postoperative PCT levels in the VAP group were significantly higher when compared with the non-VAP group during the observation period. PCT levels of >5 ng/ml had a sensitivity of 91.2% and a specificity of 82.4%, respectively. A study by Ramirez *et al* (27) examined 44 patients who received mechanical ventilation for >48 h in a medical ICU, of which 20 patients were suspected of having VAP. Based on a pathological examination, nine individuals were diagnosed with VAP, while 11 patients were assigned to the non-VAP group. On the day of VAP diagnosis confirmation, serum PCT levels and SOFA scores in the VAP group were significantly higher compared with those in the non-VAP group. The optimum cut-off value of PCT was 2.99 ng/ml, with a sensitivity of 78% and a specificity of 97%. The optimum serum PCT cut-off value in the present study was higher (5 ng/ml) when compared with the results of Oppert *et al* (17) and Ramirez *et al* (27), which may be due to the recruitment of patients from the cardiac surgery ICU, exhibiting more serious conditions and postoperative complications and higher SOFA scores.

According to a previous study, IL-6 is a cytokine secreted by giant eosinophilic cells that participates in the acute inflammatory process (28). IL-6 levels are known to increase in cardiac surgery and may be involved in the postoperative cytokine cascade (29). CRP synthesized in the liver is a nonspecific biological marker for inflammation (30). Jebali *et al* showed that serum IL-6 was not a marker for postoperative complications following cardiac surgery (23). However, the use of CRP for the diagnosis of VAP remains controversial. Póvoa *et al* (30) showed that a CRP level of >960 mg/l provided a good accuracy for VAP diagnosis, with a sensitivity of 87% and a specificity of 88%. In the present study, serum levels of CRP in the VAP and non-VAP groups did not show a statistically significant difference until three days following the diagnosis of VAP, and CRP exhibited no diagnostic value on day 1. The optimum cut-off value of IL-6 for VAP diagnosis was 70.8 pg/ml; however, the diagnostic accuracy was relatively low, with a sensitivity of 58% and a specificity of 64%. These results indicated that IL-6 and CRP were not effective for the diagnosis of VAP following cardiac surgery with CPB. Although IL-6 has been demonstrated to induce the production of PCT *in vitro* (26), no correlation was found between the levels of IL-6 and PCT.

There were a number of limitations to the present study. Firstly, the sample size was relatively small, which may have affected the diagnostic capability of PCT. Thus, further studies with a larger sample size are required to verify the results. Secondly, the present study examined only patients with early-onset VAP who had undergone elective cardiac surgery. Therefore, the results may differ from other studies that included early-onset VAP patients who were recruited from a noncardiac surgical ICU or cardiac surgery patients with late-onset VAP. Finally, the effects of surgery and CPB on serum levels of PCT remain unknown, as it was not possible to obtain the premorbid serum PCT concentrations of the patients.

In conclusion, PCT may play an important role as a biomarker in the diagnosis of early-onset VAP following cardiac surgery in clinical practice. Furthermore, serum PCT levels and SOFA scores were shown to positively correlate. However, these results require validation by further studies.

## Figures and Tables

**Table I:** Preoperative and postoperative characteristics of patients in the non-VAP and VAP groups.

Characteristic	Non-VAP group (n=59)	VAP group (n=33)	P-value
aAge (years)	47±11	50±13	NS
Gender, male/female (n)	29/30	18/15	NS
Type of surgery (n)			-
Valve surgery	32	10	-
Coronary artery bypass grafting	13	6	-
Ascending aortic surgery	8	8	-
Combined surgery	4	7	-
Others	2	2	-
Comorbidities (n)			-
Hypertension	31	10	-
Diabetes mellitus	10	4	-
Pulmonary diseases	6	4	-
Kidney disease	0	2	-
Others	6	5	-
aLVEF (%)	61±13	63±9	NS
aBaseline biological measurements			
PCT (ng/ml)	0.11±0.09	0.20±0.21	NS
IL-6 (pg/ml)	8.44±4.32	9.58±5.35	NS
CRP (ng/ml)	10.18±8.04	12.33±0.76	NS
aCPB duration (min)	118±48	156±94	NS
aAortic cross clamping time (min)	66±23	71±41	NS
aMechanical ventilation duration (days)	3±2	6±3	0.000
aLength of stay in the ICU (days)	8±6	12±9	0.001
Postoperative complication (n)	53	33	NS
Cardiovascular complication (n)	47	26	-
Pulmonary dysfunction (n)	3	4	-
Neurological complication (n)	0	2	-
Reoperation due to bleeding (n)	6	3	-
AKI requiring RRT (n)	1	3	-
aSOFA on day 1	9±3	11±2	<0.001
aSOFA on day 7	3±1	7±4	<0.001
Mortality (n)	3	7	0.02

a Results are expressed the mean ± standard deviation.

NS, not significant (P>0.05); VAP, ventilator-associated pneumonia; LVEF, left ventricle ejection fraction; PCT, procalcitonin; IL-6, interleukin-6; CRP, C-reactive protein; CPB, cardiopulmonary bypass; ICU, intensive care unit; AKI, acute kidney injury; RRT, renal replacement therapy; SOFA, sequential organ-failure assessment.

**Table II:** Diagnostic value of serum levels of PCT, IL-6 and CRP on day 1.

Protein	Optimal cut-off	AUC	Sensitivity (%)	Specificity (%)	PPV (%)	NPV (%)
PCT	5.0 ng/ml	0.87	91	71	64	93
IL-6	70.8 pg/ml	0.61	58	64	48	73
CRP	108.3 ng/ml	0.56	52	74	52	73

PCT, procalcitonin; IL-6, interleukin-6; CRP, C-reactive protein; AUC, area under the receiver operating characteristic curve; PPV, positive predictive value; NPV, negative predictive value.
